# Misrepair of DNA double-strand breaks after exposure to heavy-ion beams causes a peak in the LET–RBE relationship with respect to cell killing in DT40 cells

**DOI:** 10.1093/jrr/rrt064

**Published:** 2013-05-30

**Authors:** Mizuho Aoki-Nakano, Yoshiya Furusawa

**Affiliations:** 1 School of Allied Health Sciences, Kitasato University, 1-15-1 Kitasato, Minami-ku, Sagamihara-shi, Kanagawa 252-0373, Japan; 2 Research Center for Particle Therapy, and International Open Laboratories, National Institute of Radiological Sciences, 4-9-1 Anagawa, Inage-ku, Chiba 263-8555, Japan

**Keywords:** linear energy transfer, relative biological effectiveness, sensitivity, misrepair, heavy ion

## Abstract

To determine the radiobiological mechanisms underlying relative biological effectiveness (RBE) and the repair efficiencies of DNA double-strand breaks (DSBs) as a function of linear energy transfer (LET), we exposed cells of the chicken B-lymphocyte cell line DT40 and its DSB repair pathway-deficient derivatives to heavy-ion beams produced at the Heavy-Ion Medical Accelerator in Chiba (HIMAC) at the National Institute of Radiological Sciences (NIRS), Chiba, Japan. The relationship between LET and cell lethality was investigated in the DNA DSB repair gene knockouts Ku70^−/−^, Rad54^−/−^, and Ku70^−/−^Rad54^−/−^, and in the wild-type cells. We found that cell-cycle stage and activity of the DNA DSB repair pathways influence LET-mediated biological effects. An expected LET–RBE relationship was observed in the cells capable of DNA repair, but no peak was found in the RBE with respect to cell survival in the Ku70^−/−^Rad54^−/−^ cells or in Ku70^−/−^ cells in the G1 and early S cell-cycle phases (when no sister chromatids were present and homologous recombination could not occur). These findings suggest that the peak in RBE is caused by deficient repair of the DNA DSBs.

## INTRODUCTION

Double-strand breaks (DSBs) are considered to be the most critical among the various types of DNA damage that can be generated by ionizing radiation. In eukaryotes, two major DSB repair pathways, which differ in their requirements for DNA homology, have been identified: homologous recombination repair (HRR) and non-homologous end-joining (NHEJ). Repair of DSBs by HRR results in error-free repair of the lesion, but requires the presence of homologous sequences elsewhere in the genome [1]. The lesion is bound with a protein complex, and homologs of DNA act as a template for the missing part of the DNA [2]. Therefore, it has been suggested that the HRR pathway works only in the late S/G_2_ phase of the cell cycle [3]. DNA repair by NHEJ does not require homologous DNA [4]. In NHEJ repair, the Ku70/Ku80 heterodimer binds DNA ends and recruits the DNA-dependent protein kinase (DNA-PK) catalytic subunit (DNA-PKcs) to the site of damage [1, 4, 5]. DNA-PK joins the two ends of a DSB through a process that is largely independent of terminal DNA sequence homology. It has therefore been suggested that NHEJ works throughout the cell cycle [3, 6].

The Heavy-Ion Medical Accelerator in Chiba (HIMAC) at the National Institute of Radiological Science (NIRS) was constructed at the end of 1993, and a clinical study using carbon ions for cancer therapy has been initiated at the institute [7, 8]. HIMAC has also been operating as a multipurpose facility for both cancer treatment and biological research [9]. The high biological effectiveness of the extremely successful pioneering carbon ion therapy is mainly attributable to the relative biological effectiveness (RBE) and localized targeting of cancer cells. The RBE is one of the most important parameters in determining the biological effectiveness of ionizing radiations. RBE is a simple function of linear energy transfer (LET), which is the rate of energy deposition in the linear dimension. Heavy ions are associated with high levels of LET radiations and have been shown to be more effective than low-LET radiations in the induction of many endpoints, such as cell killing, chromosomal damage, mutation, transformation, and cell death [10–13]. In general, the RBE increases with LET, reaches a peak at around 100–200 keV/µm, and then decreases with further increase in LET, but the role of the LET–RBE relationship in many biological responses to radiation remains unknown.

In this study, we investigated chromosome damage and loss of colony-forming ability in DT40 chicken B-lymphoma cells with respect to the LET–RBE relationship.

## MATERIALS AND METHODS

### Cell culture

The chicken B-lymphoma cell line DT40 and its derivatives Ku70^−/−^, Rad54^−/−^ and Ku70^−/−^ Rad54^−/−^ were kindly provided by Dr H. Utsumi from Kyoto University. Hereafter, we have referred to them as wild-type cells, Ku70^−/−^, Rad54^−/−^, and Ku70^−/−^Rad54^−/−^, respectively. The Ku70^−/−^ cells could also be divided into two subtypes Ku70^−/−^(f) and Ku70^−/−^ (s) on the basis of radiosensitivity and cell-cycle progression, as described in the Results and Discussion sections. All the cells were incubated in a humidified atmosphere at 39.5°C and 5% CO_2_, in alpha-MEM medium supplemented with 10% fetal bovine serum, 1% chicken serum, 0.1 mg/ml streptomycin, and 100 U/ml penicillin. The doubling time for DT40 cells was 8–12 h. The cells were grown in 10-cm plastic dishes and subcultured every 2–3 d.

### Irradiation

Exponentially growing cells were collected by centrifugation, diluted to the density of ∼5 × 10^5^ cells/ml, and exposed to X-rays generated by a Pantak-320S (Shimadzu, Kyoto) at a dose rate of ∼1 Gy/min (200 kVp, using 0.5-mm aluminium and copper filters). For the heavy-ion experiments, we used a variety of ions at different accelerated energies at HIMAC (Table 1). Based on these parameters, we chose LETs in the range of 13–800 KeV/µm. No energy absorbers (for reducing possible fragmented particles) were used. Dosimetry was performed using three different methods: an ionization chamber method, a fluence measurement method using CR39 track detectors, and measurement using a silicon diode. The dosimetry, characteristics, and irradiation procedures for the heavy-ion beams have been described previously [14].

**Table 1.** Beam parameters used

Ion	In vacuum		Residual range^a^ @ IC (mm)	Absorber used^b^ (mm)	At the center of the sample		
	Beam energy (MeV/u)	Residual range (mm)			Energy (MeV/u)	Residual range (mm)	Dose-averaged LET (keV/µm)
X-ray	200 kVp						∼2.5
^12^C	290	162.9	149.5		273	147.7	13.3
^20^Ne	400	165.2	145.1		37	143.8	31
^28^Si	490	162.6	134.4		431	133.1	55
^40^Ar	550	168.0	136.4		478	135.1	85
^28^Si	135	18.89	18.9		128	17.1	130
^56^Fe	500	97.4	74.2		414	72.4	200
^40^Ar	135	16.38	5.3		55	3.43	370
^56^Fe	200	21.79	8.4		100	6.60	440
^56^Fe	200	21.79	8.4	4.6	50	2.00	800

Residual ranges are shown as equivalent to H_2_O. ^a^Values at the isocenter (IC). The values exclude the window (∼1.0 mm) of the ionization chamber. Energy is reduced by the attenuation of the scatterer, vacuum windows, and the air between the beam exit and the position of the sample. ^b^To reduce the energy and obtain higher LET, a PMMA energy degrader was inserted just upstream of the sample.

### Colony formation assay

The survival of DT40 cells was determined by their colony-forming ability, as described [15], with some modifications that included an agar-layer method. Irradiated cells were diluted to the density of ∼5 × 10^5^ cells/ml, and 0.1 ml of the cell suspension was mixed with 0.9 ml of the medium containing 0.25% agarose (Agarose LO3 TAKARA, Takara Bio Inc.), kept at 42°C. The cell suspension was spread on 6-cm petri dishes (prepared on the previous day to dry the inside of the lid) containing 5 ml of the same medium with 0.5% solidified agarose. The cells were incubated for 10 d under humid conditions with 5% CO_2_ in air at 39.5°C. Visible colonies were counted without staining under a stereomicroscope. Survival curves were generated, and radiation D_10_ values for each DT40 cell line were obtained as follows: we applied a linear exponential fit to the wild-type, Rad54^−/−^, and Ku70^−/−^Rad54^−/−^ cells, but not to the X-irradiated wild-type cells, because the survival curve data fit better with a smaller residual error compared to a linear quadratic fit. We also applied the combinational survival function SF_K_ consisting of linear and linear quadratic equations for the Ku70^−/−^ cells because they showed biphasic survival curves:

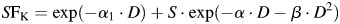

where α_1_, α, and β are parameters specific for each survival curve; *D* is an exposure dose; and *S* is the extrapolated intersection at dose 0 for the second phase.

### Fluorescence *in situ* Hybridization (FISH) analysis

Chromosomes of the irradiated cells were prematurely condensed by calyculin A (Wako Chemicals, Japan), as described elsewhere [16]. Briefly, 40 ng/ml colcemid (Gibco BRL, USA) was added to cell cultures immediately after irradiation, and cells were incubated for 2 h, then 20 nM calyculin A was added for 15 min at 39.5°C. The cells were centrifuged, resuspended in 75 mM KCl, and briefly incubated at 37°C. Freshly prepared fixative solution (methanol:acetic acid, 3:1 vol/vol) was slowly added to the cell suspension, and the cells were centrifuged, resuspended in the fixative solution, and kept at − 20°C overnight. After washing in fixative solution, chromosomes were dropped onto slides at room temperature and air-dried. Slides containing chromosomes were hybridized *in situ* with fluorescent whole-chromosome painting probe 1 (red) using Cy3-Avisin [17]. The cells were counterstained with DAPI and viewed under a fluorescence microscope. The content of aberrant cells was calculated as the ratio of cells with one or more fragments of chromosome #1 to the total number of cells analyzed.

## RESULTS

### Survival curves

**Fig. 1.**
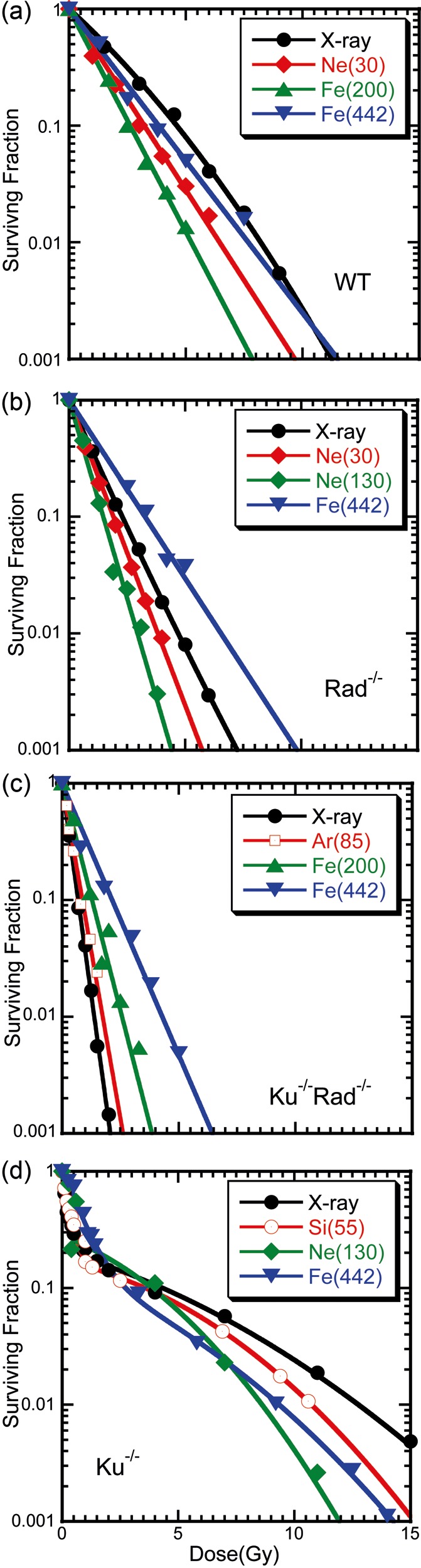
Survival curves for the (**a**) DT40 wild-type, (**b**) Rad54^−/−^, (**c**) Ku70^−/−^Rad54^−/−^, and (**d**) Ku70^−/−^ cells in different cell-cycle phases exposed to X-rays or Ne, Si, Ar or Fe ions with different LETs. The cell strains, ions and the LETs (keV/µm) are indicated.

Survival curves were generated for cells exposed to X-rays (as the reference radiation for RBEs) and to heavy-ion beams (Fig. 1). The survival curves for the wild-type cells became steeper with increasing LET up to 200 keV/µm, and the slope changed gradually at a much higher LET. The Rad54^−/−^ cells showed higher sensitivity than wild-type cells. The steepest curve was seen at 130 keV/µm among the selected LETs, and the slope was more gradual at 440 keV/µm than the curve for the cells exposed to X-rays. The curves for the Ku70^−/−^Rad54^−/−^ cells were steeper than those for all the other cell groups, and their slopes curved gradually upward with the increase in LET, which indicates that the RBE did not increase with LET. The survival curve of the Ku70^−/−^ cells showed a biphasic character. The first phase was characterized by the steepest exponential curve, while the second phase showed the most pronounced upward direction among all the curves. The curves in the first phase were similar to those of the Ku70^−/−^Rad54^−/−^ cells.

### LET and sensitivity (1/D_10_)

**Fig. 2.**
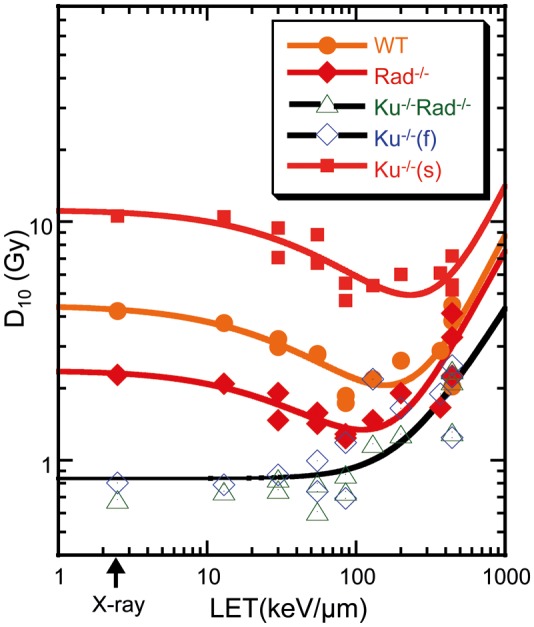
LET versus D_10_ values for cells in different knockout status (wild-type, Rad54^−/−^, Ku70^−/−^Rad54^−/−^ or Ku70^−/−^ ). Ku70^−/−^ cells at different cell cycle stages were divided into two subtypes: Ku70^−/−^(f) (in the G_1_/early-S phase), or Ku70^−/−^(s) (in the late-S/G_2_ phase). Plots of Ku70^−/−^Rad54^−/−^ and Ku70^−/−^(f) are fitted together. Each mark corresponds to inversed D_10_ values from a single experiment.

The D_10_ values (as reciprocal values of the radiosensitivity) were calculated from the survival data for DT40 cells exposed to different heavy-ion beams, and the relationship between LET and D_10_ for each cell type was plotted (Fig. 2). The biphasic survival curve for the Ku70^−/−^ cell suggests the presence of two subpopulations with different radiosensitivities, representing different cell-cycle groups (with or without sister chromatids in the cell) that differed in their radiosensitivity. The Ku70^−/−^(f) cells were in the first steep phase of the survival curve, and the Ku70^−/−^(s) cells, in the second gradual phase. To obtain D_10_s for the second phase, the intersection (S in equation) that the extrapolated value at dose 0 from the data at higher was shifted up to 1, and obtain the 10% survival dose. The Ku70^−/−^(s) cells showed more than 10 times higher D_10_ values, and the Rad54^−/−^ cells showed approximately three times higher D_10_ values, than those for the Ku70^−/−^Rad54^−/−^ or Ku70^−/−^(f) cells at low LET. The D_10_ values for the wild-type cells were between those for the Ku70^−/−^(s) and the Ku70^−/−^Rad54^−/−^ or Ku70^−/−^(f) cells. Compared to the results for low LET, in the high-LET region, the D_10_ values decreased by ∼2.5 times for both Ku70^−/−^(s) and wild-type cells; for the Rad54^−/−^ cells, the reduction was approximately two times or lesser. No reductions were detected in the D_10_ values for the Ku70^−/−^Rad54^−/−^ or the Ku70^−/−^(f) cells.

### Chromosome fragmentation

**Fig. 3.**
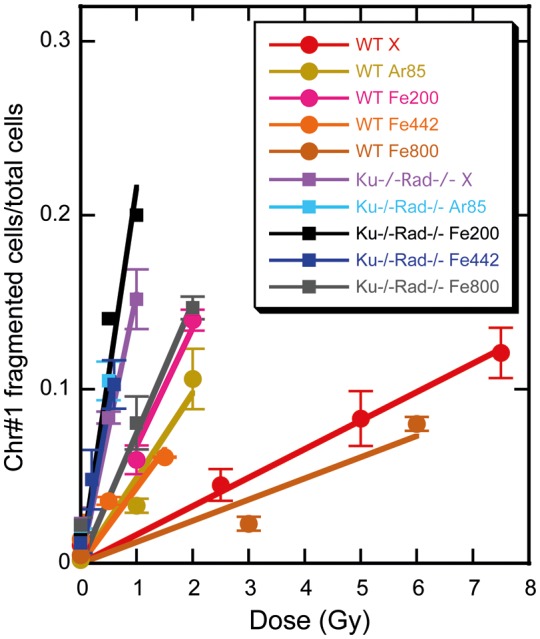
Dose–response for the frequency of chromosome #1 fragmentation in the wild-type and Ku70^−/−^Rad54^−/−^ cells after exposure to X-rays, Ar ions or Fe ions with different LETs (in keV/µm). Values with error bars are calculated from at least three experiments.

**Fig. 4.**
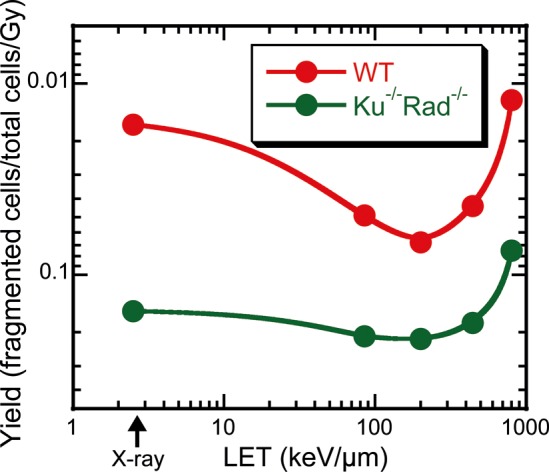
Induction yields of chromosome #1 fragmentation per Gy in wild-type or Ku70^−/−^Rad54^−/−^ cells exposed to heavy ions, as a function of LET. Note that the vertical axis is inverted to be seen as the same as the D_10_ spectrum in Fig. 2. The lines represent a simple smooth fit.

Figure 3 shows the data for chromosome fragmentation as an initial DNA response to damage for the Ku70^−/−^Rad54^−/−^ cells and the wild-type cells exposed to: X-rays, Ar ions at 85 keV/µm, or Fe ions at 200, 440 or 800 keV/µm. The induction of chromosome #1 fragmentation in both cell types showed a linear correlation with the dose, up to a dose of ∼200 keV/µm, but then decreased with LET (Fig. 4).

## DISCUSSION

The survival curve for the Ku70^−/−^ cells could be divided into two phases, the sensitive and the resistant phases. The extrapolated value of the survival fraction for Ku70^−/−^(s) to dose 0 from the higher dose was approximately 0.2, and this value corresponded to the G2 phase population in the total cells from flow-cytometric analysis (data not shown). A similar result was reported by Utsumi *et al.* [18]. The Ku70^−/−^Rad54^−/−^ cells, defective in both the HRR and NHEJ repair pathways, and the Ku70^−/−^ cells at first phase (Ku70^−/−^(f)), showed the steepest survival curves; the HRR-defective Rad54^−/−^ cells were next in radiosensitivity, followed by the wild-type cells (Fig. 1). Thus, the Ku70^−/−^(s) cells, which contain homologous DNA and are therefore suitable for HRR, were the most resistant among the DT40 cells. Because the possible repair system in the Ku70^−/−^ cells depends on the cell cycle, they demonstrated biphasic radiosensitivity with increase in the ionizing radiation dose (Fig. 1), indicating the presence of two distinct cell populations [1]. In the sensitive phase of the survival curves, the Ku70^−/−^(f) cells are in the G1 and early S phases of the cell cycle, when neither HRR nor NHEJ can operate, and therefore show similar sensitivity to that of the Ku70^−/−^Rad54^−/−^ cells. However, in the resistant phase, the Ku70^−/−^(s) cells are in the S–G2 phase of the cell cycle, when HRR is used for DSB repair, and therefore are more resistant than the wild-type cells. These observations suggest that HRR acts more effectively in the absence of Ku70 in the late S and G_2_ phases. In contrast to the Ku70^−/−^ cells, the Rad54^−/−^ cells did not show increased radioresistance in the late S and G_2_ phases [1], which implies that NHEJ is a relatively stable DSB repair pathway during the cell cycle, which cannot repair DSB perfectly. HRR is efficient in the late S and G_2_ phases of the cell cycle because of the requirement for sister chromatids as repair templates, and is down-regulated during the G_0_, G_1_, and early S phases [1]. HRR, the *Rad54*-dependent recombination repair pathway, may function only after a pair of sister chromatids is generated by DNA replication.

The process of DNA repair by HRR or NHEJ depends not only on the phase of the cell cycle but also on the complexity of the damaged DNA ends. The effects of clustered DNA damage induced by the high-LET ionizing radiation accounts for the increase in cell lethality, because clustered DNA damage is difficult to repair [19]. Less complex DSBs are repaired preferentially by NHEJ, which is the dominant pathway for DSB repair in mammalian cells during all stages of the cell cycle, whereas more complex DSBs containing multiple damaged sites that cannot be repaired by NHEJ are repaired by HRR when homologous DNA strands become available in the late S to G_2_ phases. Furthermore, HRR and NHEJ may compete passively, with the repair outcome depending, for example, on whether HRR or NHEJ proteins bind first to the broken ends, and/or the availability of an homologous repair template. Irreversible binding of Ku70 to DSB ends would block access to HRR proteins [20]. Therefore, the contribution of HRR decreases when Ku70 is present, as is the case in the wild-type cells.

The RBEs for repairable cells showed a typical LET-dependence, i.e. RBE increase, peaking at 100–200 keV/μm, and then decrease with further increase in LET. However, the RBE for irreparable cells did not show a peak but simply decreased with LET above 200 keV/μm, providing an initial yield of DSBs in cells [21]. The increase in the RBE with LET is explained by accumulation of irreparable or long-persisting DSBs as the primary lesions leading to cell death and chromosome anomalies [22], i.e. yields of irreparable and clustered DSBs [23]. The rapid decrease in RBE at LET above 200 keV/μm, on the other hand, is thought to be a consequence of the excessive energy deposit in the cell, i.e. energy in excess of the level required for causing a single radiological event [24, 25]. Alternatively, the decrease in RBE in the very high LET range may be due to the increasing fraction of surviving cells (cells not traversed by heavy ions) with LET at an equivalent dose [26].

We suggest that misrepair of DSBs causes the peak in the LET–RBE relationship in cell killing. The HRR system is error-free, whereas the NHEJ system is typically an error-prone system in which nucleotide alternations are tolerated at the site of rejoining. Bogomazova *et al.* proposed that DSB misrejoining by NHEJ was the main cause of chromatid exchange formation in human embryonic stem cells (hESC) and differentiated cells [27]. Wild-type cells were less resistant than Ku70^−/−^(s) cells when the cells were in the late-S/G_2_ phase, and the HRR system was used for DSB repair, because of the competition between HRR and NHEJ. We hypothesize that the competition is caused by the NHEJ proteins that are quickly recruited to the damaged site in the wild-type cells, thus preventing the error-free HRR system from operating. However, HRR works well in the Ku70^−/−^(s) cells and repairs the damage site without error, which results in better cell survival than that seen in wild-type cells.

When the LET–RBE relationship in Fig. 1 is analyzed in terms of LET and sensitivity (Fig. 2.), the spectra can be split on four different fitting lines. The sensitivity is mostly low for the irreparable Ku70^−/−^Rad54^−/−^ and Ku70^−/−^(f) cells and simply increases with LET. The curve shift indicates increasing resistance for the Rad54^−/−^, wild-type, and Ku70^−/−^(s) cells, with increase in repair potential. The D_10_ values for X-rays and for ion beams at 100–200 keV/µm (Fig. 2) are listed in Table 2. *Rad54* protein can increase cell survival by approximately three times (2.27/0.80); *rad54* and *ku70* proteins in the wild-type cells by five times (4.23/0.80); and the *rad54-*positive without *ku70* in Ku70^−/−^ cells could survive more than 10 times (10.57/0.80) those D_10_ values. These values correspond to the repair potential of the cells with each genotype at the peak LET (100–200 keV/µm). The repair potentials of *rad54* (the Ku70^−/−^(s) cells), *ku70* with *rad54* (the wild-type cells), and *ku70* without *rad54* (the Ku70^−/−^(f) cells) were reduced to 40% (4.5/10.57), 40% (1.8/4.23), and 60% (1.3/2.27), respectively, at high LET (ion beam) compared to low LET (X-rays).

**Table 2:** D_10_ values and the ratios for X-rays and ion beams at 100–200 keV/µm

Cell type	Functional	D_10_ (Gy)		Ratio of D_10_	
	Gene	X-rays^a^	ion beam^b^	X-rays	ion beam
Ku70^−/−^Rad54^−/−^ or Ku70 ^−/−^(f)	none	0.80	1.0	1.0	1.0
Rad54^−/−^	ku70	2.27	1.3	2.8	1.3
Wild-type	ku70^+^ rad54	4.23	1.8	5.3	1.8
Ku70^−/−^(s)	rad54	10.57	4.5	13.2	4.5

^a^Obtained from survival curves. ^b^Approximate D_10_s at the 100–200 keV/µm read from Fig. 2.

Similar results were also obtained for the relationship between LET and the frequency of chromosome #1 fragmentation (Fig. 4). Inverted values of the sensitivity corresponded to D_10_s. The frequencies of the cells with the fragmented chromosome #1 after X-ray irradiation were approximately 10 times different between the wild-type and Ku70^−/−^Rad54^−/−^ cells. This difference was twice as high as for D_10_ in survival curves. We also could see a slight but clear increase in the slope (which appears as a decrease in Fig. 2) at 100–200 keV/µm for the wild-type and Ku70^−/−^Rad54^−/−^ cells. This small increase in fragmentation was not accompanied by an increase in observed cell death for Ku70^−/−^Rad54^−/−^, which suggests the activity of some other repair systems for supporting cell survival or the presence of chromosome aberrations that do not have lethal effects [27].

We can conclude that the repair is responsible for the peak in the LET–RBE on the basis of the difference in the LET–RBE spectrum between cell strains that use different repair systems in the cell cycle phase but have the same genetic background.
